# Predictive value of bone metabolism markers in the progression of diabetic kidney disease: a cross-sectional study

**DOI:** 10.3389/fendo.2024.1489676

**Published:** 2024-11-04

**Authors:** Yi Kang, Qian Jin, Mengqi Zhou, Zirong Li, Huijuan Zheng, Danwen Li, Weijing Liu, Yaoxian Wang, Jie Lv

**Affiliations:** ^1^ Dongzhimen Hospital, Beijing University of Chinese Medicine, Beijing, China; ^2^ Renal Research Institution of Beijing University of Chinese Medicine, Beijing, China; ^3^ Graduate School of Beijing University of Chinese Medicine, Beijing, China; ^4^ Department of Traditional Chinese Medicine, Beijing Puren Hospital, Beijing, China

**Keywords:** type 2 diabetes mellitus, diabetic kidney disease, bone metabolism markers, predictive value, progression

## Abstract

**Objective:**

This study aimed to investigate the relationship between bone metabolism markers, including serum klotho, fibroblast growth factor 23 (FGF23), 25(OH)D3, iPTH, calcium (Ca), and PHOS and the progression of diabetic kidney disease (DKD) in patients with type 2 diabetes mellitus (T2DM). Additionally, the predictive value of these markers for DKD progression was evaluated.

**Methods:**

This study involved 126 patients with T2DM between May 2021 and March 2023. DKD staging was assessed based on urinary protein excretion rates and estimated glomerular filtration rate (eGFR). The study evaluated serum concentrations of klotho, FGF23, 25(OH)D3, iPTH, Ca and PHOS across various stages and examined their relationships with clinical parameters. Receiver operating characteristic (ROC) curve analysis was utilized to determine the predictive accuracy of these bone metabolism markers for DKD. Multivariate linear and logistic regression analyses identified risk factors linked to DKD severity.

**Results:**

Among the 126 participants, 30 had non-DKD with normal proteinuria, while 96 had DKD, categorized as 31 with stage III DKD (microproteinuria), 34 with stage IV DKD, and 31 with stage V DKD (massive proteinuria). With advancing DKD from stage III to V, levels of klotho, 25(OH)D3, and Ca decreased significantly, whereas FGF23, iPTH and PHOS levels increased markedly. Klotho is significantly positively correlated with eGFR (*r* = 0.285, *P* = 0.001.) and negative correlations with serum creatinine (Scr) and UACR (*r* = -0.255, *P* = 0.004; *r* = -0.260, *P* = 0.011). FGF23 was positively related to systolic blood pressure (SBP) (*r* = 0.224, *P* = 0.012), but negatively with eGFR (*r* = -0.294, *P* = 0.001). Additionally, 25(OH)D3 exhibited significant negative correlations with several adverse clinical biomarkers, and both iPTH, Ca and PHOS were strongly associated with DKD progression (*P*<0.05). ROC analysis showed high predictive accuracy for DKD using these bone metabolism markers, with a combined area under the curve (AUC) of 0.846. Multivariate logistic regression analysis reinforced the significance of these markers in DKD progression.

**Conclusion:**

Bone metabolism markers, such as klotho, FGF23, 25(OH)D3, iPTH, Ca and PHOS are intricately linked to DKD progression and may function as valuable predictive biomarkers.

## 1 Introduction

Diabetic kidney disease (DKD) presents a substantial public health concern due to its increasing prevalence and the absence of effective interventions to prevent its progression. As a complication arising from diabetes, DKD inflicts damage on various renal components, including the glomeruli, tubules, interstitium, and vasculature (1). Clinically, DKD manifests through a spectrum of proteinuria and a continuous decline in renal function, eventually progressing to end-stage renal disease (ESRD). Although renal biopsy is the definitive diagnostic method for DKD, its invasive nature limits its widespread application in clinical settings. Consequently, clinicians rely on key indicators such as the urinary albumin excretion rate and estimated glomerular filtration rate (eGFR) to diagnose and monitor DKD progression. However, these parameters are subject to numerous influencing factors, leading to insufficient sensitivity in detecting renal injury. Therefore, the quest for more reliable biomarkers for DKD screening and prediction remains ongoing (2). Historically, DKD research has predominantly viewed the kidney as an isolated organ, concentrating on its molecular pathological mechanisms. However, emerging evidence underscores the significance of inter-organ communication in disease pathogenesis, with the “bone-kidney axis” becoming a pivotal concept in DKD studies. Factors such as hyperglycemia, accumulation of advanced glycation end products (AGEs), and the progression of chronic kidney disease (CKD) contribute to disruptions in calcium-phosphorus metabolism, abnormal parathyroid hormone (PTH) levels, and impaired bone mineralization, all of which can precipitate bone disease (3). Diabetes is an independent risk factor for skeletal health (4). In the context of DKD, the interactions among key bone regulatory factors (FGF23, klotho, and iPTH) are further disrupted, accelerating the progression of bone disease (5). The skeletal system, now recognized as an endocrine organ, secretes bone-derived hormones that are intricately involved in key pathogenic processes of DKD, such as energy metabolism (6), insulin resistance (7), inflammation (8), and epithelial-mesenchymal transition (9). Moreover, disruptions in bone metabolism may further accelerate DKD progression (10).

Previous research on mineral and bone disorders in CKD has largely concentrated on the later stages, such as end-stage renal disease (ESRD) and dialysis, where abnormalities in mineral and bone metabolism are more pronounced (11). However, emerging studies have highlighted that bone-derived hormone abnormalities can occur early in CKD (12, 13). Identifying these abnormalities early is vital for disease management. For example, a prospective study involving T2DM patients found that elevated FGF23 levels independently predict all-cause mortality and are closely linked with an increased risk of progressing to ESRD (14–16). The interaction between bone and kidney function is crucial, as FGF23, secreted mainly by osteoblasts and osteocytes (17), relies on renal Klotho protein for its biological effects (18). FGF23 regulates renal sodium, calcium, and phosphate balance, vitamin D metabolism, immune-inflammatory responses, and the renin-angiotensin-aldosterone system (RAAS), and affects Klotho, angiotensin-converting enzyme 2, and erythropoietin expression (19, 20). Klotho is also a potential biomarker for early DKD detection (21). These insights suggest that bone metabolism disturbances significantly impact DKD progression and renal function. Investigating the communication between bone and kidney may clarify DKD pathogenesis (22).FGF23 and klotho are involved in phosphate and vitamin D metabolism and calcium-phosphate balance in CKD patients. This study examines the correlation between serum levels of klotho, FGF23, 25(OH)D3, iPTH, calcium (Ca) and PHOS with DKD progression in clinical patients, evaluating the predictive value of these bone metabolism markers. By analyzing different DKD stages in T2DM patients, the study aims to offer new reference markers for the early diagnosis and intervention of DKD.

## 2 Methods

### 2.1 Study design and population

**Figure 1:**
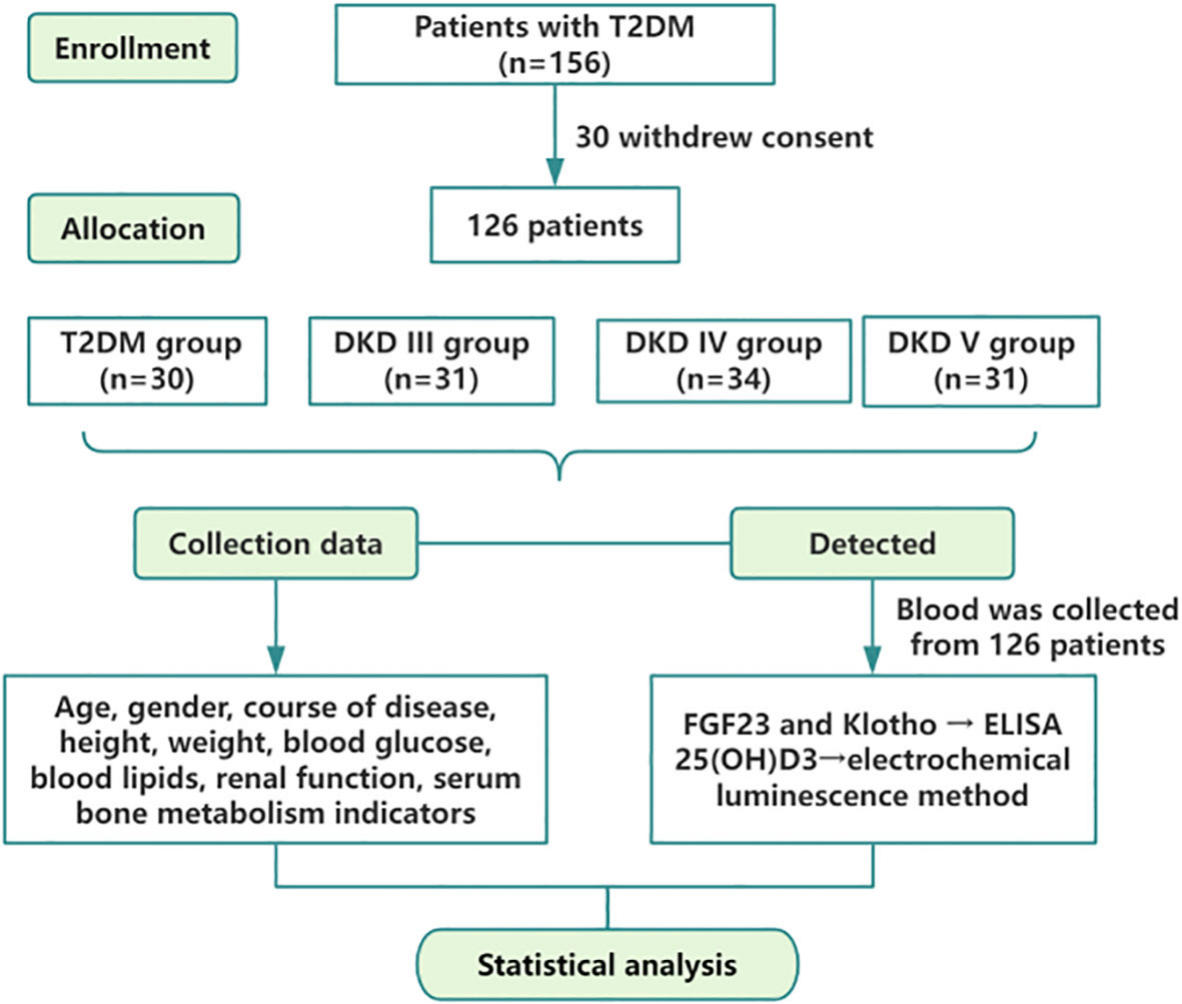
Diagram of the study design.

This study encompassed patients diagnosed with type 2 diabetes mellitus (T2DM) and DKD who were admitted to Dongzhimen Hospital, Dongcheng Campus, Beijing University of Chinese Medicine, from May 2021 to March 2023. The study was sanctioned by the Ethics Committee of Dongzhimen Hospital, Beijing University of Chinese Medicine (2022DZMEC-062-03), and all participants provided written informed consent. The flowchart is depicted in **Figure 1**.

### 2.2 Inclusion and exclusion criteria

The inclusion criteria were (1):age between 30 and 90 years (2);diagnosis of T2DM conforms to the “Chinese Guidelines for the Prevention and Treatment of Type 2 Diabetes” (2020 edition) (23); diagnosis of DKD based on the 2007 National Kidney Foundation (NKF-KD/OQI) guidelines (24); the 2020 Clinical Practice Guideline for the Evaluation and Management of CKD by the Kidney Disease: Improving Global Outcomes (KDIGO) organization (25) and the 2021 Chinese Clinical Guidelines for the Diagnosis and Treatment of Diabetic Kidney Disease (26). Staging criteria followed the 2007 NKF-KD/OQI guidelines (24) and the Mogensen staging criteria (27).

The DKD staging criteria are defined as follows: Stage III DKD: Urinary albumin-to-creatinine ratio (UACR) consistently ranges from 30-300 mg/g, with a eGFR remaining near normal or slightly elevated. Stage IV DKD: UACR exceeds 300 mg/g, or 24-hour urine protein levels are greater than 0.5 g, accompanied by a significant reduction in eGFR; Stage V DKD (end-stage renal failure, uremia stage): eGFR drops below 15 ml/min/1.73 m². Patients are classified based on proteinuria as follows: Non-DKD patients have normal proteinuria with UACR less than 30 mg/g; DKD patients are divided into two groups: the microproteinuria group with UACR ranging from 30-300 mg/g, and the massive proteinuria group with UACR exceeding 300 mg/g.

Exclusion criteria included (1): patients who had used medications such as active vitamin D, calcitonin, bisphosphonates, or other agents affecting calcium and phosphorus metabolism within the past six months; (2) patients who had undergone parathyroidectomy; (3) patients who had received dialysis treatment; (4) those with primary or secondary kidney diseases; (5) patients with urinary tract infections or other acute or chronic inflammatory conditions; (6) patients with liver function abnormalities, autoimmune diseases, malignant tumors, hematologic disorders, or mental illnesses; and (7) patients who had experienced trauma, surgery, or psychological stress within the last six months.

### 2.3 Data collection and measurement

#### 2.3.1 Participant characteristics

Patient clinical data, including age, gender, height, weight, systolic blood pressure, and diastolic blood pressure, were collected. Body mass index (BMI) was calculated as height/weight².

#### 2.3.2 Laboratory tests

Fasting venous blood samples were collected from all participants on the second morning after hospital admission. The samples were analyzed using a Mindray CL1000i automatic chemiluminescence analyzer for 25(OH)D3 and iPTH. Additionally, an AU680 automatic biochemical analyzer (Beckman Coulter, USA) was used to measure serum Ca, PHOS, glucose (GLU), total protein (TP), urea (UREA), serum creatinine (CREA), uric acid (UA), 24-hour urine protein quantification (24-UTP), high-density lipoprotein cholesterol (HDL-C), and low-density lipoprotein cholesterol (LDL-C). The estimated glomerular filtration rate (eGFR) was calculated using the CKD-EPI formula.

For females:


$$\begin{array}{l} \text{eGFR}=144\times {\left(\frac{\frac{\text{Serum Creatinine}}{88.4}}{0.7}\right)}^{-0.329}\times 0.993^{\text{Age}}\text{(if Serum Creatinine }\leq \;61.88\text{ μmol}/\text{L)}\\ \text{eGFR}=144\times {\left(\frac{\frac{\text{Serum Creatinine}}{88.4}}{0.7}\right)}^{-1.209}\times 0.993^{\text{Age}}\text{(if Serum Creatinine>}61.88\text{ μmol}/\text{L)} \end{array}$$

For Males:


$$\begin{array}{l} \text{eGFR}=141\times {\left(\frac{\frac{\text{Serum Creatinine}}{88.4}}{0.9}\right)}^{-0.411}\times 0.993^{\text{Age}}\text{(if Serum Creatinine }\leq \;79.56\text{ μmol}/\text{L)}\\ \text{eGFR}=141\times {\left(\frac{\frac{\text{Serum Creatinine}}{88.4}}{0.9}\right)}^{-1.209}\times 0.993^{\text{Age}}\text{(if Serum Creatinine>}79.56\text{ μmol}/\text{L)} \end{array}$$

#### 2.3.3 Detection of bone metabolism-related biomarkers

On the second day of hospitalization, fasting venous blood samples (2 mL) were collected from each participant in the morning. After standing at room temperature for 30 minutes, the samples were centrifuged at 3000 rpm for 10 minutes at 4°C. The resulting serum was carefully separated, aliquoted into labeled EP tubes, and stored at -80°C for future analysis. Intact FGF23 and klotho levels were measured using enzyme-linked immunosorbent assay (ELISA) kits provided by Elabscience (Hubei, China).

### 2.4 Statistical methods

SPSS 26.0 was employed for statistical analysis. Quantitative data following a normal distribution were presented as mean ± standard deviation (x¯ ± s), whereas non-normally distributed data were shown as median with interquartile range. Categorical variables were reported as frequencies and percentages (%). For normally distributed quantitative data, comparisons between groups were conducted using one-way analysis of variance. For non-normally distributed quantitative data, comparisons between groups were performed using non-parametric tests, specifically the Kruskal-Wallis test. Chi-square tests were used for categorical data analysis. The Bonferroni correction was applied to adjust p-values for multiple comparisons within groups. Pearson or Spearman rank correlation tests assessed correlations. Multiple linear regression evaluated the independent associations of eGFR with serum levels of FGF23, klotho, 25(OH)D3, iPTH, Ca and PHOS. Multinomial logistic regression was used to examine the link between DKD severity and bone metabolism-related markers. *P*<0.05 was deemed statistically significant.

## 3 Results

### 3.1 Clinical characteristics of study participants

A total of 126 T2DM patients participated in this study, including 80 males and 46 females, with a mean age of 59.50 ± 11.29 years. The cohort consisted of 30 patients with T2DM alone (normal proteinuria [NP]) and 96 patients with DKD, divided into 31 with DKD stage III (microproteinuria [MP]), 34 with DKD stage IV, and 31 with DKD stage V (massive proteinuria [MAP]). The findings reveal no notable differences across the four groups regarding age, gender, BMI, DBP, and lipid profiles. Conversely, significant increases in SBP and UA levels were observed in the DKD IV and V groups. Additionally, a marked decrease in TP levels was noted. The DKD V group demonstrated a considerable reduction in FPG levels. Analyses of Scr and eGFR indicated that Scr levels were significantly elevated in both the DKD IV and V groups. Simultaneously, eGFR exhibited a substantial decline (*P* < 0.001), pointing to a pronounced worsening of renal function. Moreover, UACR and 24-UTP displayed a rise (*P* < 0.001), suggesting a close association between disease progression and the severity of proteinuria (**Table 1**).

**Table 1:** Baseline Characteristics of Participants.

Variables	T2DM (n = 30)	DKD III(n=31)	DKD IV(n=34)BD268	DKD V(n=31)	*P*
	Normal proteinuria (n=30)	Microproteinuria (n=31)	Massive proteinuria (n =65)		
Age (years)	58.80 ± 11.62	58.87 ± 11.58	60.41 ± 11.57	59.81 ± 10.85	0.93
Male (%)	17 (56.67)	17 (54.84)	21 (61.76)	25 (80.65)	0.136
Female (%)	13 (43.33)	14 (45.16)	13 (38.23)	6 (19.35)	
BMI (kg/m^2^)	24.6 (22.63,26.91)	25.54 (23.41,29.9)	25.56 (22.82,27.91)	26.05 (24.17,29.50)	0.922
SBP (mmHg)	134.80 ± 18.36	132.10 ± 18.85	141.38 ± 19.96^#^	146.90 ± 17.26^*#^	**0.010**
DBP (mmHg)	81.03 ± 14.37	78.13 ± 14.44	78.56 ± 10.90	75.81 ± 9.71	0.444
FPG (mmol/L)	10.83 (8.38,15.86)	10.07(7.06,14.49)	8.83 (6.97,12.20)	6.64 (6.00,9.15)*	**0.002**
TG (mmol/L)	1.70 (1.15,2.76)	1.59(1.05,2.53)	1.90 (1.24,3.38)	1.40 (1.05,2.00)	0.115
LDL-C (mmol/L)	2.56 (2.07,3.67)	2.78(1.99,4.04)	3.14 (2.02,3.74)	2.35 (1.66,3.06)	0.206
HDL-C (mmol/L)	1.18 ± 0.27	1.21 ± 0.30	1.28 ± 0.39	1.14 ± 0.35	0.379
TP (g/L)	71.35 (66.25,76.55)	73.30(65.80,75.30)	63.50 (59.25,66.35)**	60.50 (56.30,66.60)**^##^	**<0.001**
ALP (U/L)	85.09 ± 18.54	77.17 ± 24.61	87.05 ± 31.80	85.14 ± 23.93	0.422
UA (μmol/L)	342.05 (284.35,410.48)	295.40(266.80,407.40)	396.00 (329.70,434.43)	451.80 (395.90,502.40)*^##^	**<0.001**
Scr (μmol/L)	63.25 (53.40,83.15)	65.40(60.70,73.00)	138.00 (102.80,248.80)**^##^	488.50 (406.40,635.30)**^##▴▴^	**<0.001**
eGFR (ml/min/1.73m^2^)	98.34 (83.83,106.88)	97.25(85.28,116.87)	39.73 (22.57,54.25)**^##^	9.86 (7.50,12.03)**^##▴▴^	**<0.001**
UACR (mg/g)	15.00 (10.00,16.25)	150.00(100.00,180.00)**	800.00 (800.00,1500.00)**^##^	————–	**<0.001**
24h-UTP (mg)	87.00 (55.00,131.50)	174.00(143.00,246.00)*	3754.00 (1567.25,6861.00)**^##^	4466.00 (2831.00,6697.00)**^##^	**<0.001**

BMI, body mass index; SBP, systolic blood pressure; DBP, diastolic blood pressure; FPG, Fasting Plasma Glucose; TG, triglycerides; LDL-C, low-density lipoprotein cholesterol; HDL-C, high-density lipoprotein cholesterol; TP, total protein; ALP, alkaline phosphatase; UA, serum uric acid; Scr, serum Scrtinine; eGFR, estimated glomerular filtration rate; UACR, urine albumin to Scrtinine ratio; 24h-UTP, 24-hour Urine Total Protein. Compared with the T2DM group, ^*^ *p*<0.05, ^**^ *p*<0.001; compared with the DKD III group, ^#^ *p*<0.05, ^##^ *p*<0.001; compared with the DKD IV group, ^▲^ *p*<0.05, ^▲▲^ *p*<0.001. Bold values indicate P<0.05.

### 3.2 Changes in bone metabolism markers across different stages of DKD and T2DM

**Figure 2:**
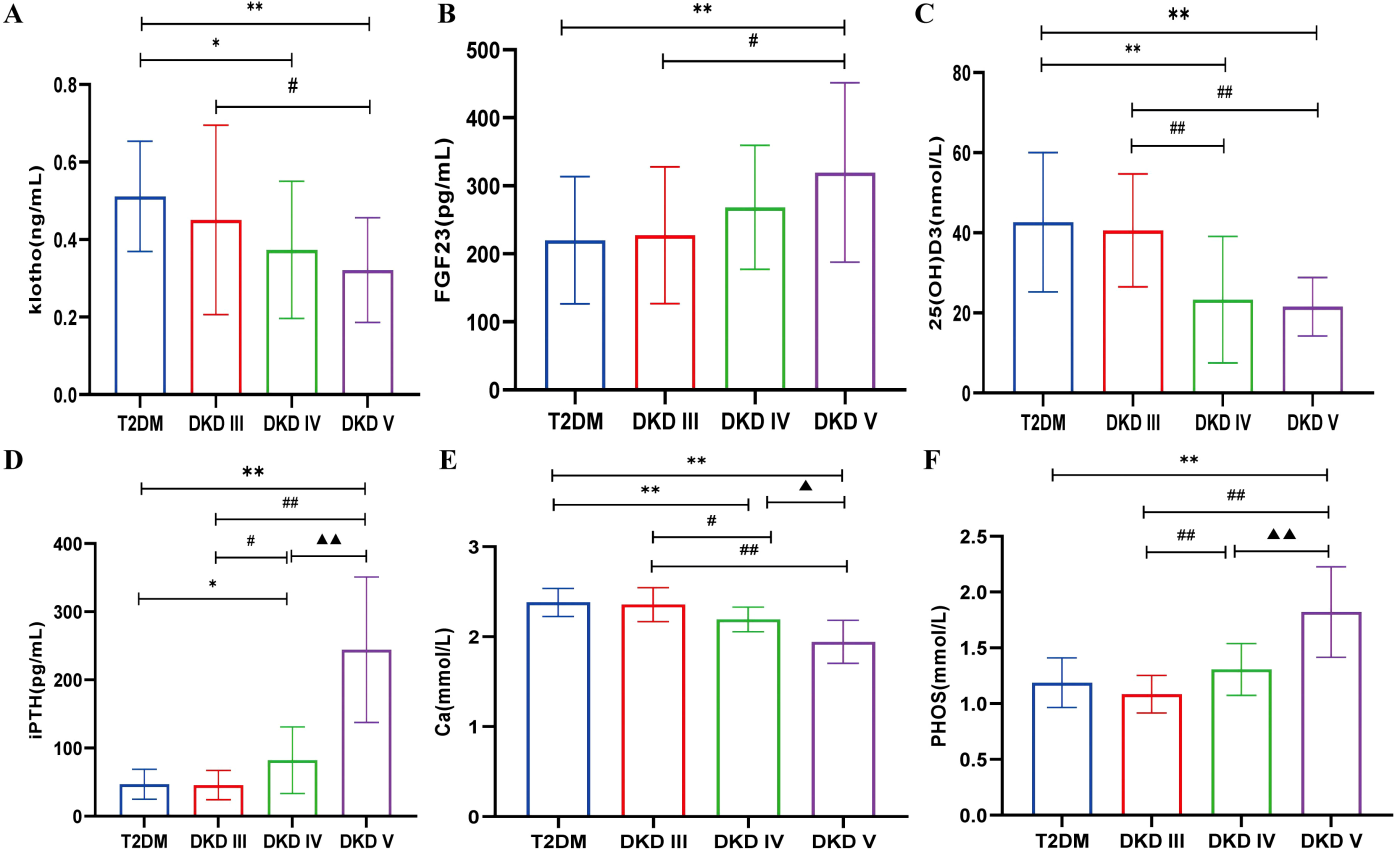
The levels of bone metabolism markers among different stages of DKD and T2DM group. **(A)** serum klotho. **(B)** serum FGF23. **(C)** serum 25(OH)D3. **(D)** serum iPTH. **(E)** serum Ca. **(F)** serum PHOS. Compared with the T2DM group, ^*^ *p*<0.05, ^**^ *p*<0.001; compared with the DKD III group, ^#^ *p*<0.05, ^##^ *p*<0.001; compared with the DKD IV group, ^▲^ *p*<0.05, ^▲▲^ *p*<0.001.

Compared with the T2DM group, klotho levels were significantly lower in DKD stage IV (0.373 ± 0.177) and stage V (0.321 ± 0.135) (*P*<0.05). Similarly, klotho levels in DKD stage V showed a declining trend compared to stage III (0.451 ± 0.244) (*P*<0.05) (**Figure 2A**). Compared with the T2DM group (219.95 ± 93.48) and DKD stage III (227.23 ± 100.45), FGF23 levels were significantly elevated in DKD stage V (319.54 ± 131.93) (*P*<0.05) (**Figure 2B**). Compared with the T2DM group, 25(OH)D3 levels were significantly lower in DKD stage IV (23.30 ± 15.80) and stage V (21.54 ± 7.31) (*P*<0.05). A similar downward trend was observed in DKD stages IV and V compared to stage III (40.58 ± 14.10) (**Figure 2C**). Compared with the T2DM group (46.85 ± 22.08), iPTH levels were significantly higher in DKD stage IV (82.06 ± 48.76) and stage V (244.05 ± 106.66) (*P*<0.05). Compared to DKD stage III, iPTH levels also showed an increasing trend in DKD stages IV and V, with stage V levels significantly higher than stage IV (*P*<0.05) (**Figure 2D**). Compared with the T2DM group (2.38 ± 0.16), Ca levels were significantly lower in DKD stage IV (2.38 ± 0.16) and stage V (1.94 ± 0.24) (*P*<0.05). A similar decreasing trend was observed in DKD stages IV and V compared to stage III (2.36 ± 0.19), with Ca levels in stage V significantly lower than in stage IV (*P*<0.05) (**Figure 2E**). The PHOS level in the DKD V group (1.82 ± 0.40) was significantly higher compared to the T2DM group (1.19 ± 0.22), DKD III group (1.08 ± 0.17), and DKD IV group (1.31 ± 0.23) (*P* < 0.05). Additionally, compared to the DKD III group, the PHOS level in the DKD IV group showed an increasing trend (*P* < 0.05) (**Figure 2F**).

### 3.3 Changes in bone metabolism markers among different proteinuria groups

**Figure 3:**
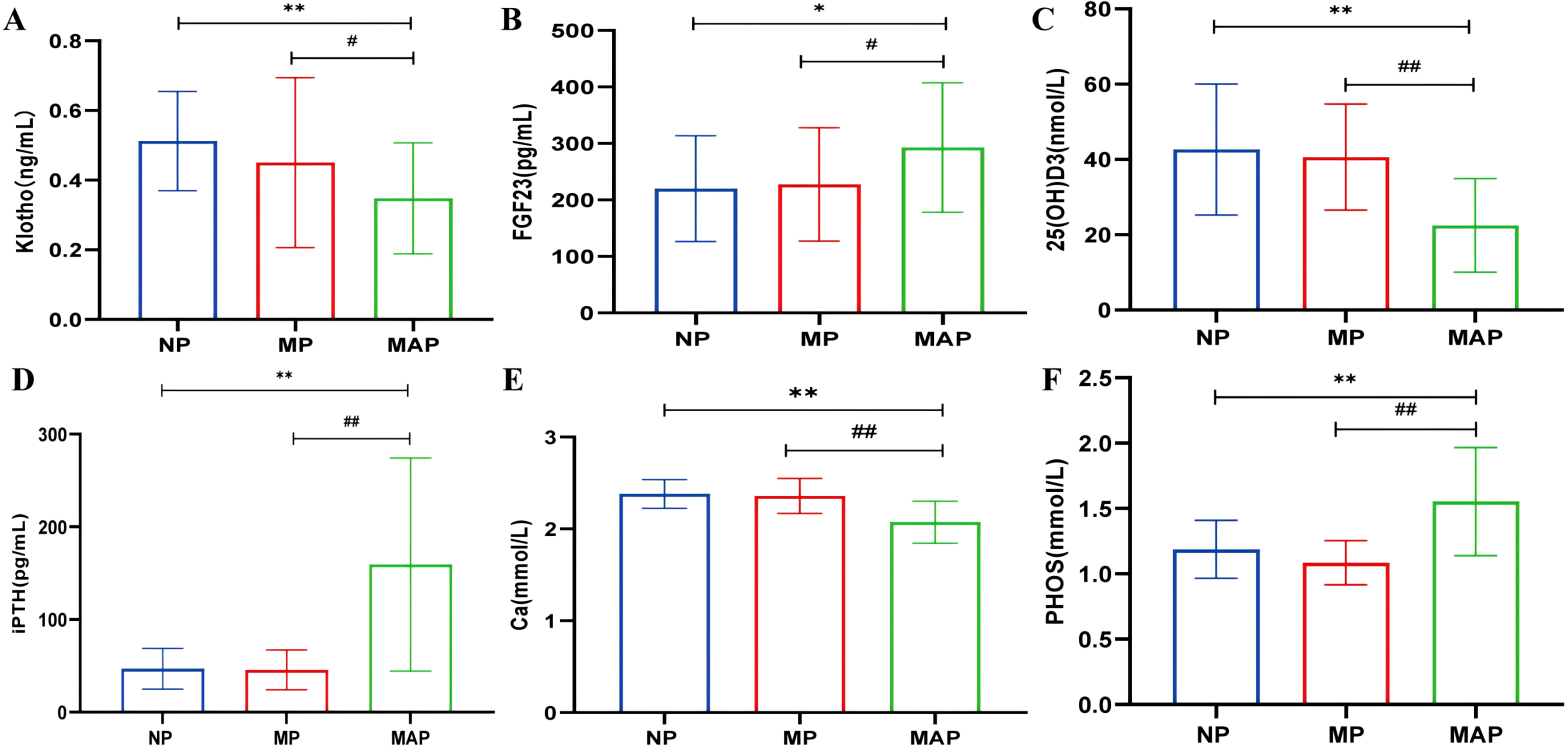
The levels of bone metabolism markers among different proteinuria groups. **(A)** serum klotho. **(B)** serum FGF23. **(C)** serum 25(OH)D3. **(D)** serum iPTH. **(E)** serum Ca. **(F)** serum PHOS. Compared with the NP group, ^*^ *p*<0.05, ^**^ *p*<0.001; compared with the MP group, ^#^ *p*<0.05, ^##^ *p*<0.001.

Patients were categorized into NP, MP, and MAP groups based on proteinuria levels. There were no significant differences in bone metabolism markers between the NP and MP groups. However, significant differences were observed in the MAP group compared to both NP and MP groups, indicating that alterations in bone metabolism occur predominantly in the later stages of DKD with massive proteinuria. klotho, 25(OH)D3, and Ca levels were lower, while FGF23, iPTH and PHOS levels were higher in the MAP group compared to the NP and MP groups (*P*<0.05) (**Figures 3A–F**).

### 3.4 Correlation analysis between bone metabolism markers and clinical indicators in DKD patients

**Figure 4:**
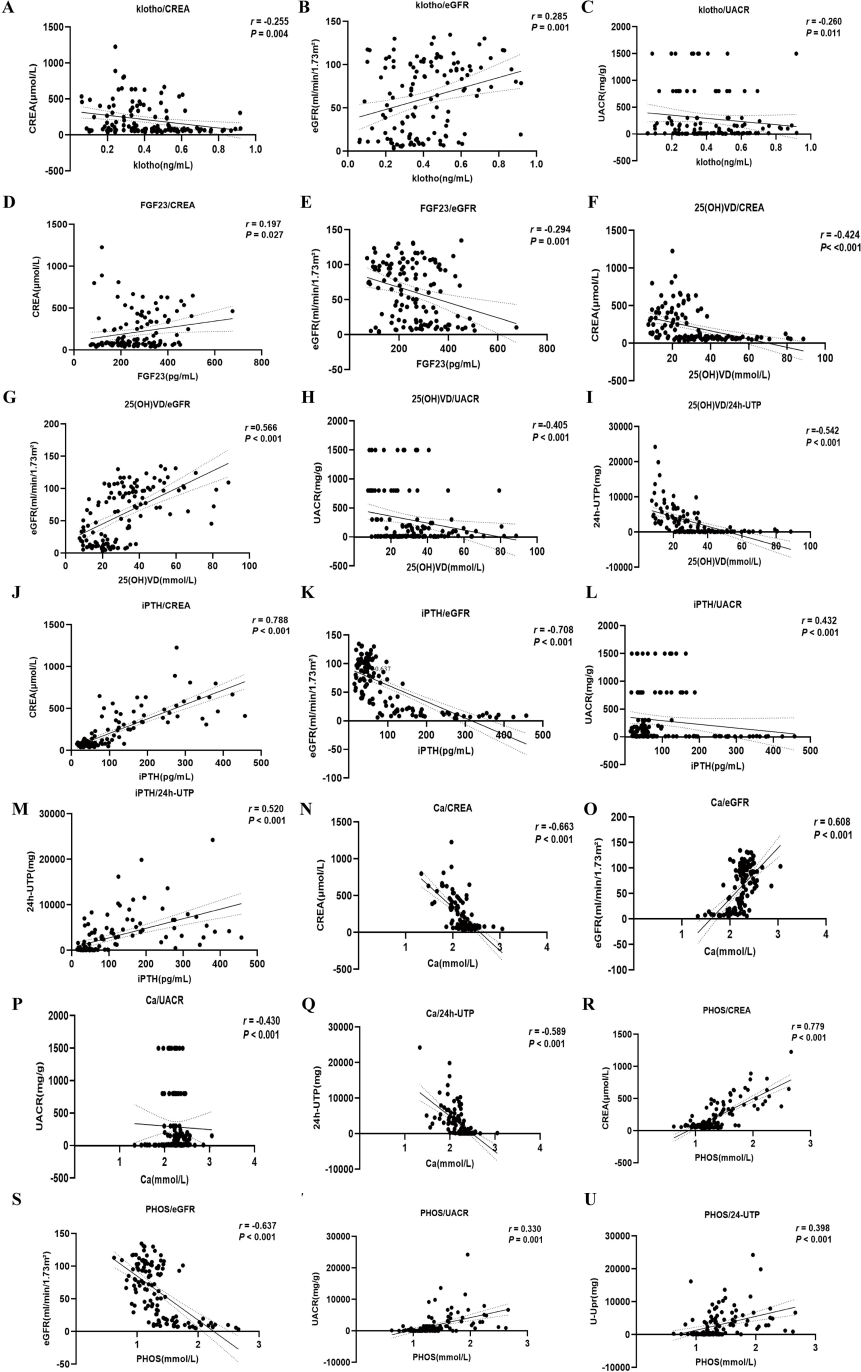
Correlation analysis of bone metabolism-related indexes and clinical biomarkers. **(A-C)** serum Klotho. **(D, E)** serum FGF23. **(F-I)** serum 25(OH)D3. **(J-M)** serum iPTH. **(N-Q)** serum Ca. **(R-U)** serum PHOS.

A correlation analysis involving 126 patients identified significant relationships between bone metabolism indicators and clinical parameters. Klotho levels showed a positive correlation with eGFR (*r* = 0.285, *P* < 0.001) and negative correlations with Scr and UACR (*r* = -0.255, *P* = 0.004; *r* = -0.260, *P* = 0.011), suggesting that lower klotho levels may link to worsening renal function (**Figures 4A–C**). FGF23 levels positively correlated with SBP (*r* = 0.224, *P* = 0.012) and negatively with eGFR (*r* = -0.294, *P* = 0.001), indicating that higher FGF23 levels might be associated with increased blood pressure and declining renal function (**Figures 4D, E**). The level of 25(OH)D3 negatively correlated with SBP, ALP, UA, Scr, UACR, and 24h-UTP (*P* < 0.05), while positively correlating with TP and eGFR (**Figures 4F–I**). iPTH levels positively correlated with UA, Scr, UACR, and 24h-UTP (*r* = 0.328, *P* < 0.001; *r* = 0.788, *P* < 0.001; *r* = 0.432, *P* < 0.001; *r* = 0.520, *P* < 0.001) and negatively with eGFR (*r* = -0.708, *P* < 0.001), implying that elevated iPTH levels are closely tied to renal function decline and metabolic issues (**Figures 4J–M**). Ca levels exhibited positive correlations with eGFR (*r* = 0.608, *P* < 0.001) and negative correlations with UA (*r* = -0.315, *P* < 0.001), Scr (*r* = -0.663, *P* < 0.001), UACR (*r* = -0.430, *P* < 0.001), and 24h-UTP (*r* = -0.589, *P* < 0.001), suggesting that fluctuations in Ca levels may indicate the severity of renal impairment and proteinuria (**Figures 4N–Q**). PHOS levels were negatively correlated with eGFR (*r* = -0.637, *P* < 0.001) and positively with UA (*r* = 0.364, *P* < 0.001), Scr (*r* = 0.779, *P* < 0.001), UACR (*r* = 0.330, *P* = 0.001), and 24h-UTP (*r* = 0.398, *P* < 0.001), indicating that PHOS levels notably rise during renal function deterioration (**Figures 4R–U**) (**Table 2**).

**Table 2:** Correlation analysis between bone metabolism markers and clinical indicators.

Variables	klotho		FGF23		25 (OH)D3		iPTH		Ca		PHOS	
	*r*	*P*	*r*	*P*	*r*	*P*	*r*	*P*	*r*	*P*	*r*	*P*
Age	-0.016	0.862	0.010	0.914	0.048	0.591	0.039	0.666	-0.035	0.697	-0.213	**0.017**
Gender	-0.018	0.838	-0.116	0.197	0.096	0.287	-0.245	**0.006**	0.258	**0.003**	-0.109	0.226
BMI(kg/m^2^)	0.026	0.775	-0.055	0.538	-0.122	0.174	0.159	0.076	-0.063	0.480	0.127	0.156
SBP(mmHg)	-0.038	0.675	0.224	**0.012**	-0.308	**<0.001**	0.247	**0.005**	-0.172	0.054	0.242	**0.006**
DBP(mmHg)	0.058	0.516	0.075	0.401	-0.032	0.721	-0.100	0.263	0.128	0.155	0.020	0.826
FPG(mmol/L)	0.129	0.151	-0.045	0.616	0.083	0.357	-0.266	**0.003**	0.198	**0.026**	-0.331	**<0.001**
TG(mmol/L))	-0.064	0.483	-0.067	0.463	-0.110	0.228	-0.177	0.053	0.178	0.051	-0.088	0.339
LDL-C(mmol/L)	0.029	0.757	-0.136	0.143	-0.046	0.619	-0.134	0.148	0.158	0.087	-0.083	0.373
HDL-C(mmol/L)	0.027	0.765	-0.046	0.615	0.03	0.747	-0.033	0.716	0.16	0.079	0.011	0.904
TP(g/L)	0.107	0.234	-0.186	0.038	0.362	**<0.001**	-0.387	**<0.001**	0.673	**<0.001**	-0.287	0.001
ALP(U/L)	-0.09	0.317	0.117	0.193	-0.178	**0.046**	0.082	0.359	0.024	0.792	0.067	0.457
UA(μmol/L)	-0.102	0.258	0.181	**0.043**	-0.329	**<0.001**	0.328	**<0.001**	-0.315	**<0.001**	0.364	<0.001
Scr(μmol/L)	-0.255	**0.004**	0.197	**0.027**	-0.424	**<0.001**	0.788	**<0.001**	-0.663	**<0.001**	0.779	**<0.001**
eGFR(ml/min/1.73m^2^)	0.285	**0.001**	-0.294	**0.001**	0.566	**<0.001**	-0.708	**<0.001**	0.608	**<0.001**	-0.637	**<0.001**
UACR(mg/g)	-0.260	**0.011**	0.144	0.165	-0.405	**<0.001**	0.432	**<0.001**	-0.430	**<0.001**	0.330	**0.001**
24h-UTP(mg)	-0.170	0.062	0.111	0.225	-0.542	**<0.001**	0.520	**<0.001**	-0.589	**<0.001**	0.398	**<0.001**

BMI, body mass index; SBP, systolic blood pressure; DBP, diastolic blood pressure; FPG, Fasting Plasma Glucose; TG, triglycerides; LDL-C, low-density lipoprotein cholesterol; HDL-C, high-density lipoprotein cholesterol; TP, total protein; ALP, alkaline phosphatase; UA, serum uric acid; Scr, serum Scrtinine; eGFR, estimated glomerular filtration rate; UACR, urine albumin to Scrtinine ratio; 24h-UTP, 24-hour Urine Total Protein. Bold values indicate P<0.05.

### 3.5 Clinical predictive value of bone metabolism indexes for DKD severity

**Figure 5:**
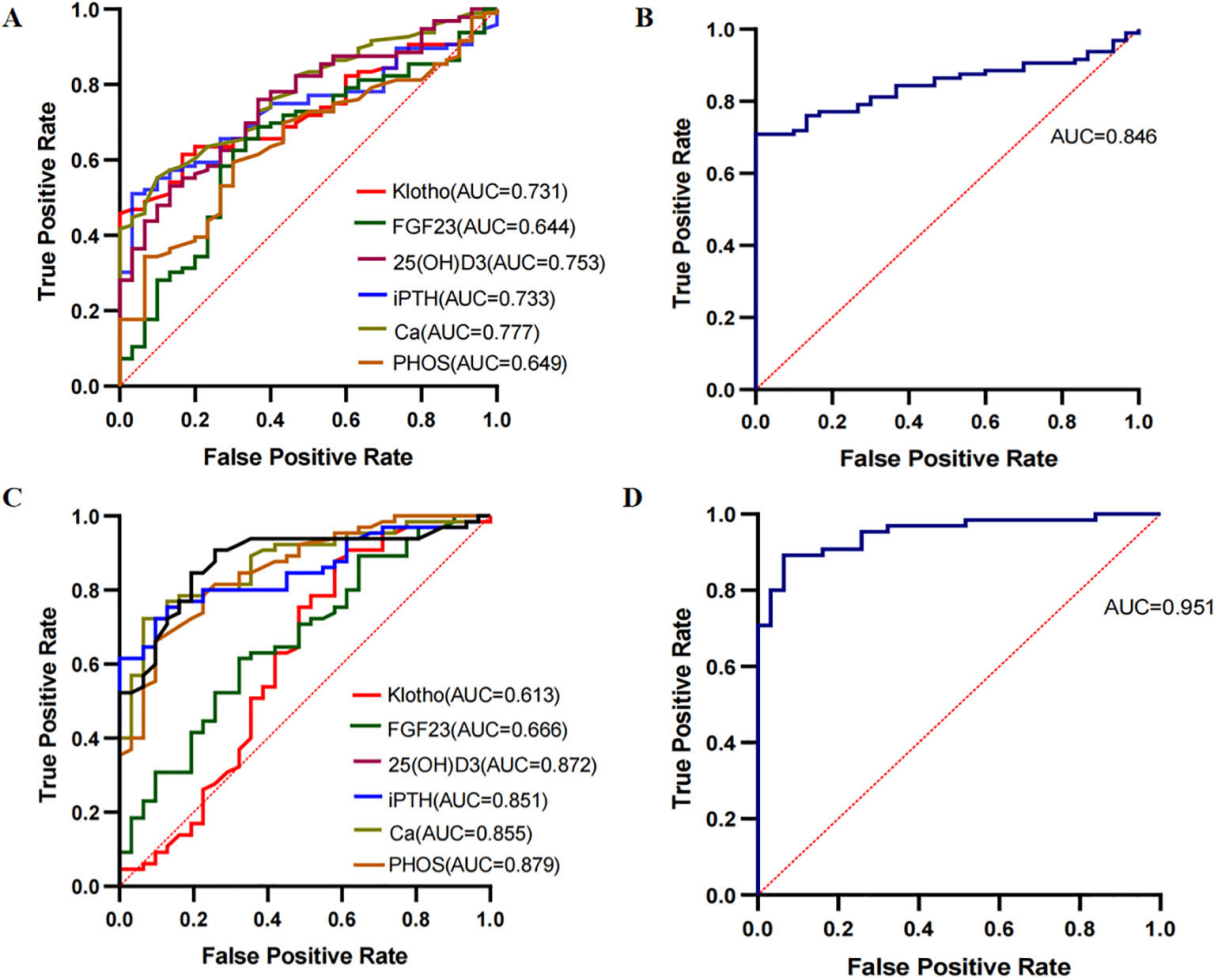
ROC curves for predicting DKD using bone metabolism markers. **(A, B)** ROC curves for klotho, FGF23, 25(OH)D3, iPTH, Ca and PHOS predicting NDKD and DKD groups. **(C, D)** ROC curves for klotho, FGF23, 25(OH)D3, iPTH, Ca and PHOS predicting MP and MAP groups.

The DKD group was merged into non-DKD (NDKD, n = 30) and DKD (n = 96) groups. ROC curves were plotted to explore the clinical predictive value of bone metabolism markers for DKD (**Figure 5**). Klotho, FGF23, 25(OH)D3, iPTH, Ca and PHOS all showed predictive value for DKD in T2DM patients, with AUCs greater than 0.7 for all except FGF23 and PHOS (**Figure 5A**). The optimal cutoffs and corresponding sensitivity and specificity for each marker are shown in **Table 3**. The combined AUC for the six bone metabolism markers predicting DKD was 0.846 (*P*<0.001) (**Figure 5B**). DKD stages IV and V were combined into the MAP group based on proteinuria levels. ROC curves for the MP group (n=31) and MAP group (n=65) were plotted to analyze the AUC for predicting MP and MAP in DKD patients. The AUC for predicting MP and MAP with bone metabolism markers was 0.951 (*P*<0.001) (**Figure 5C**). Among these, 25(OH)D3, iPTH, Ca and PHOS had AUCs greater than 0.85. The optimal cutoffs and corresponding sensitivity and specificity for each marker are detailed in **Table 3**. The combined AUC for predicting DKD was 0.951 (*P*<0.001) (**Figure 5D**).

**Table 3:** Predictive value of bone metabolism markers for DKD.

	Variables	AUC	Cut-off	Sensitivity	Specificity
NDKD group VS DKD group	klotho	0.731	< 0.331	0.458	1.000
	FGF23	0.644	> 236.6	0.625	0.700
	25(OH)D3	0.753	< 26.62	0.531	0.867
	iPTH	0.733	> 78.78	0.510	0.967
	Ca	0.777	< 2.215	0.552	0.900
	PHOS	0.649	>1.245	0.594	0.700
MP group VS MAP group	klotho	0.613	< 0.525	0.877	0.419
	FGF23	0.666	> 267.2	0.615	0.677
	25(OH)D3	0.872	< 26.16	0.723	0.936
	iPTH	0.851	> 69.35	0.723	0.903
	Ca	0.855	< 2.205	0.662	0.903
	PHOS	0.879	>1.210	0.846	0.806

### 3.6 Simple linear regression analysis of eGFR with clinical and bone metabolism indicators

The eGFR was used as the dependent variable, with Age, Gender, BMI, SBP, DBP, FPG, TP, TG, LDL-C, HDL-C, ALP, UA, Scr, klotho, FGF23, 25(OH)D3, iPTH, Ca and PHOS as independent variables (**Table 4**). Simple linear regression analysis showed that eGFR was positively correlated with FPG, TP, Klotho, 25(OH)D3, and Ca, and negatively correlated with SBP, UA, Scr, UACR, FGF23, iPTH and PHOS (*P*<0.05).

**Table 4:** Linear regression analysis of independent correlated factors.

Index	B (95%CI)	*t*	*P*
Age	-0.461(-1.104,0.182)	-1.419	0.158
Gender(Male=1,Female=2)			0.068
BMI	0.103(-1.885,2.091)	0.103	0.918
SBP	-0.698(-1.056,-0.34)	-3.86	<0.001
DBP	0.554(-0.024,1.133)	1.896	0.060
FPG	2.073(0.767,3.38)	3.141	0.002
TP	2.596(1.831,3.36)	6.72	<0.001
TG	2.177(-1.793,6.148)	1.086	0.280
LDL-C	6.886(-0.27,14.042)	1.906	0.059
HDL-C	-4.283(-26.383,17.817)	-0.384	0.702
ALP	-0.133(-0.42,0.154)	-0.916	0.361
UA	-0.216(-0.281,-0.152)	-6.625	<0.001
Scr	-0.148(-0.168,-0.128)	-14.561	<0.001
UACR	-0.046(-0.056,-0.036)	-8.974	<0.001
klotho	61.165(24.65,97.68)	3.315	0.001
FGF23	-0.109(-0.172,-0.046)	-3.427	0.001
25(OH)D3	1.37(1.015,1.724)	7.653	<0.001
iPTH	-0.289(-0.34,-0.237)	-11.163	<0.001
Ca	99.713(76.539,122.886)	8.517	<0.001
PHOS	-67.388(-81.9, -52.877)	-9.191	<0.001

### 3.7 Multinomial logistic regression analysis of disease severity and bone metabolism markers in DKD patients

The multinomial logistic regression analysis was performed with DKD severity as the dependent variable (DKD III=3, DKD IV=4, DKD V=5), and SBP, FPG, ALP, TP, klotho, FGF23, 25(OH)D3, iPTH, Ca and PHOS as independent variables. It is aimed to assess the trends of bone metabolism markers across different DKD severity groups. In comparison to the T2DM group, klotho levels were significantly lower in patients with DKD IV and V group. Even after adjusting for different models, particularly Model 2 and Model 3, the reduction in klotho levels remained strongly linked to DKD progression, signifying that as DKD worsens, klotho levels notably decrease. Similarly, FGF23 levels in the DKD V group were significantly elevated compared to the T2DM group, with *P* < 0.05 across all models (Model 1, Model 2, and Model 3), underscoring the marked rise in FGF23 levels in advanced DKD. In the DKD IV group, the levels of 25(OH)D3 were significantly lower than those in the T2DM group in Model 1 and Model 2 (*P* < 0.05), suggesting that 25(OH)D3 levels decrease significantly relative to the control group as DKD progresses to stage IV, while DKD V did not show statistical significance. When comparing to DKD s III group, iPTH levels in DKD V group were significantly elevated across all models (*P* < 0.05), showing that iPTH levels increase as DKD progresses. Ca levels were significantly reduced in DKD IV and V group when compared to both the T2DM group and DKD III group, highlighting a strong negative correlation between Ca levels and DKD severity (*P* < 0.05). PHOS levels were significantly higher in DKD III and V group compared to the T2DM group and were also elevated in DKD IV and V group when compared to DKD III group (*P* < 0.05). Notably, these bone metabolism markers varied in their timing of changes, with PHOS showing significant differences as early as DKD III group, while FGF23 levels only differed significantly in DKD V group, suggesting that disturbances in bone metabolism play a significant role in the pathological progression of DKD (**Table 5**).

**Table 5:** Multiple logistic regression analysis of the relationship between DKD severity and bone metabolism.

Dependent variables	Independent variables	Model 1			Model 2			Model 3		
		β	Wald	*P*	β	Wald	*P*	β	Wald	*P*
T2DM group*	klotho	-2.113	2.216	0.137	-2.104	2.079	0.149	-2.807	3.119	0.077
DKD III group	FGF23	0.001	0.206	0.650	0.003	0.727	0.394	0.004	1.196	0.274
	25(OH)D3	-0.021	1.32	0.251	-0.025	1.818	0.178	-0.029	2.05	0.152
	iPTH	-0.016	1.246	0.264	-0.024	2.366	0.124	-0.024	1.915	0.166
	Ca	-0.202	0.013	0.911	0.179	0.009	0.925	0.712	0.076	0.783
	PHOS	-3.800	5.299	**0.021**	-4.462	6.139	**0.013**	-4.815	6.586	**0.010**
T2DM group*	klotho	-4.053	4.506	**0.034**	-4.105	4.558	**0.033**	-4.082	4.157	**0.041**
DKD IV group	FGF23	0.004	1.240	0.265	0.005	1.6	0.206	0.005	1.352	0.245
	25(OH)D3	-0.068	4.915	**0.027**	-0.066	4.509	**0.034**	-0.062	3.443	0.064
	iPTH	0.003	0.061	0.804	0.002	0.026	0.871	0.006	0.149	0.700
	Ca	-8.247	7.299	**0.007**	-8.821	7.342	**0.007**	-6.590	2.736	0.098
	PHOS	2.606	1.685	0.194	2.936	1.751	0.186	2.880	1.541	0.214
T2DM group*	klotho	-12.68	3.963	**0.047**	-17.052	3.962	**0.047**	-21.163	3.119	0.077
DKD V group	FGF23	0.018	5.005	**0.025**	0.021	4.97	**0.026**	0.024	4.088	**0.043**
	25(OH)D3	0.113	1.771	0.183	0.139	2.003	0.157	0.247	2.153	0.142
	iPTH	0.035	3.214	0.073	0.044	3.034	0.082	0.060	3.263	0.071
	Ca	-19.48	7.213	**0.007**	-21.886	6.091	**0.014**	-20.49	4.77	**0.029**
	PHOS	7.146	5.460	**0.019**	7.893	5.785	**0.016**	12.796	4.057	**0.044**
DKD III group*	klotho	-1.940	1.074	0.300	-2.001	1.087	0.297	-1.275	0.374	0.541
DKD IV group	FGF23	0.003	0.587	0.444	0.002	0.335	0.563	0.001	0.068	0.795
	25(OH)D3	-0.047	2.415	0.120	-0.041	1.753	0.185	-0.034	0.981	0.322
	iPTH	0.019	1.420	0.233	0.027	2.212	0.137	0.029	2.366	0.124
	Ca	-8.045	6.922	**0.009**	-9.00	7.351	**0.007**	-7.302	3.498	0.061
	PHOS	6.406	8.818	**0.003**	7.398	9.458	**0.002**	7.695	9.081	**0.003**
DKD III group*	klotho	-10.567	2.765	0.096	-14.949	3.048	0.081	-18.355	2.341	0.126
DKD V group	FGF23	0.017	4.290	**0.038**	0.018	3.747	0.053	0.020	2.912	0.088
	25(OH)D3	0.134	2.495	0.114	0.165	2.799	0.094	0.276	2.679	0.102
	iPTH	0.051	5.688	**0.017**	0.068	6.198	**0.013**	0.084	5.616	**0.018**
	Ca	-19.278	7.064	**0.008**	-22.065	6.158	**0.013**	-21.202	5.125	**0.024**
	PHOS	10.945	11.987	**0.001**	12.356	13.059	**0.000**	17.611	7.463	**0.006**

*reference group.

Model 1, not adjusted; Model 2, adjusted Model 1+SBP and FPG; Model 3, adjusted Model 2+ALP and TP. Bold values indicate P<0.05.

## 4 Discussion

This study examined changes in bone metabolism markers in patients with T2DM and DKD, focusing on the association of klotho, FGF23, 25(OH)D3, iPTH, Ca and PHOS levels with the severity of DKD. Furthermore, this study also assesses their predictive value for the progression of DKD. It analyzes the significance of bone metabolism markers both separately and in combination for the DKD and NDKD groups, as well as for DKD patients with microproteinuria and massive proteinuria. The results indicate that as DKD advances from stage III to V, klotho, calcium, and 25(OH)D3 levels show a significant decline, whereas FGF23, iPTH, and PHOS levels experience a marked increase. Multiple regression analyses further confirmed that these bone metabolism markers are closely related to DKD progression, suggesting their potential clinical utility in the diagnosis and prognosis of DKD.

### 4.1 Relationship between bone metabolism dysregulation and DKD progression

**Figure 6:**
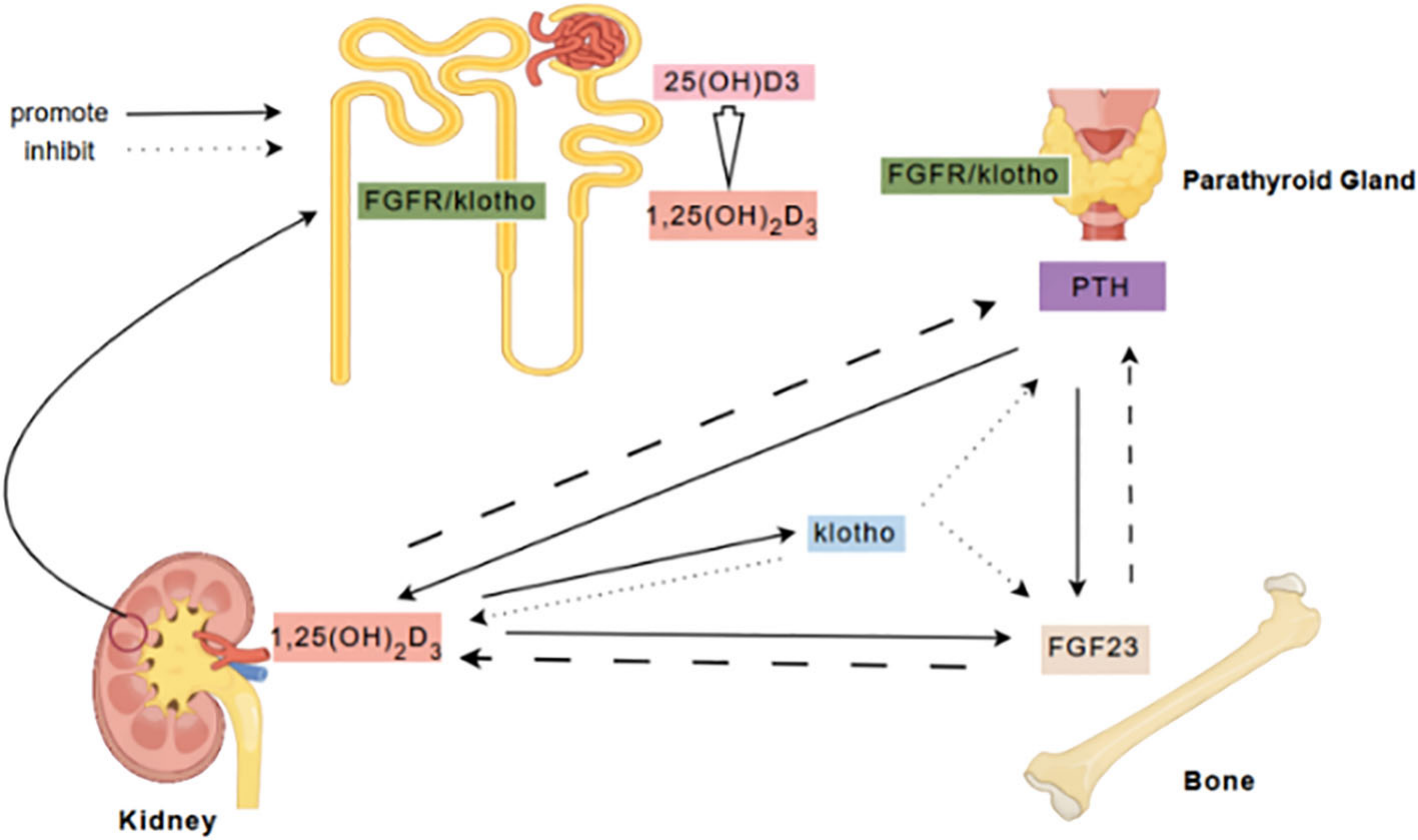
FGF23/Klotho Axis in Bone-Kidney Communication. FGF23, secreted by bones, primarily binds to the Klotho/FGFR complex in the kidneys and parathyroid glands to inhibit the secretion of 1,25(OH)2D3 and PTH, and regulate renal phosphate excretion. Klotho, acting as a co-receptor for FGF23, modulates phosphate metabolism by suppressing 1,25(OH)2D3 synthesis and promoting phosphate excretion. In the kidneys, 25(OH)D3 is converted to 1,25(OH)2D3, which enhances intestinal calcium absorption and inhibits PTH secretion through negative feedback. PTH, secreted by the parathyroid glands, acts on bones and kidneys to stimulate bone resorption and renal calcium reabsorption. These factors maintain calcium-phosphate balance and bone mineral metabolism through complex feedback and feedforward mechanisms.

The communication between bone and kidney is a complex process involving mutual regulation among different molecules (**Figure 6**). FGF23 is a phosphate-regulating hormone primarily secreted by bone cells. As a crucial molecule in the communication between bone and kidney, FGF23 collaborates with active vitamin D and intact parathyroid hormone to regulate calcium and phosphate homeostasis (18), and are involved in the pathological processes of insulin resistance, inflammation, fibrosis, and podocyte injury in DKD (8, 28–30). FGF23 levels increase significantly with DKD progression, and elevated FGF23 levels during kidney function decline are a compensatory response to phosphate retention, consistent with its known role in CKD (31). Chronic low-grade inflammation is a major pathogenic mechanism of DKD (32), and the inflammatory response can further increase the expression of FGF23 (33). In addition, excessive elevation of FGF23 may also lead to complications such as left ventricular hypertrophy and cardiovascular events (34). Thus, FGF23 serves not only as a marker of bone metabolism dysregulation but also as a potential predictor of cardiovascular risk. Our study found that FGF23 is positively correlated with SBP, further suggesting that elevated FGF23 levels may be associated with increased blood pressure and worsening kidney function.

Klotho is a single-pass transmembrane protein predominantly expressed in the kidneys and parathyroid glands (19, 35). Apart from acting as a co-ligand for FGF23 to regulate PHOS, Ca, 1,25(OH)2D3, and iPTH metabolism, klotho also protects the kidneys through various mechanisms, including antioxidant stress responses, anti-inflammatory effects, anti-fibrosis, induction of autophagy, inhibition of apoptosis and aging, and maintaining renal endothelial cell integrity (20, 36). The kidneys are the primary organ for klotho gene expression (37). Our previous studies have confirmed that in the context of DKD, renal aging is accelerated, and factors such as hyperglycemia, inflammation, oxidative stress, and hypertension can induce the downregulation of klotho expression (38). Plasma klotho levels are reduced in DKD patients, and klotho levels are negatively correlated with the annual decline rate of eGFR and the occurrence of albuminuria (39, 40). Klotho levels begin to decline early in CKD and decrease further as the disease progresses (41), and considered biological markers that aid in the early diagnosis of DKD (42). Our study observed a significant decline in klotho levels with DKD progression, with klotho levels in all DKD groups being lower than those in the T2DM group, and decreasing further from stage III to stage V. We found a significant positive correlation between klotho and eGFR, which supports its role as a marker of worsening renal function.

1,25(OH)2D3 facilitates the absorption of Ca and PHOS in the intestines and regulates Ca metabolism in bone tissue (43). 1,25(OH)2D3 participates in renal protection in DKD through pathways such as inducing autophagy, alleviating oxidative stress, anti-inflammation, anti-fibrosis, and inhibiting RAS activation (44–47). Previous studies have found that vitamin D deficiency is more pronounced in diabetic patients compared to healthy controls and is more likely to lead to diabetic microvascular complications (48–51). Vitamin D deficiency is a significant risk factor for DKD compared to diabetic patients without renal impairment (52–54). A retrospective cohort study involving 161 patients diagnosed with DKD via biopsy further confirmed that patients with lower serum 25(OH)D3 levels had more severe glomerular lesions and higher scores of tubular atrophy and interstitial fibrosis (55). Our study also observed a progressive decline in 25(OH)D3 levels with DKD progression, particularly marked in DKD stages IV and V. According to the diagnostic criteria for vitamin D deficiency (<75 nmol/L) set by the Italian Society of Clinical Endocrinology and the American Association of Clinical Endocrinologists (56), vitamin D deficiency was already present in stage III DKD patients. As the main circulating form of vitamin D, a decrease in 25(OH)D3 levels may reflect a decline in renal synthetic function. Low levels of 25(OH)D3 were significantly negatively correlated with several adverse clinical biomarkers, further supporting its protective role in DKD.

iPTH, as a key regulator of calcium-phosphate metabolism, directly affects blood calcium levels and enhances calcium absorption by influencing 1,25(OH)2D3 conversion. Our study found that iPTH levels significantly increase with DKD progression, particularly in stage V. Elevated iPTH often reflects the presence of CKD-MBD, which may be more common in DKD patients (57). Increased iPTH could be a compensatory response to hypocalcemia and hyperphosphatemia, but may also lead to excessive bone turnover and cardiovascular calcification (58).

The changes in serum Ca and PHOS levels also exhibited significant trends in this study. As DKD progresses from stage III to stage V, Ca levels gradually decrease while serum PHOS levels gradually increase, particularly in stage V of DKD. Hypocalcemia and hyperphosphatemia may result from hyperparathyroidism, vitamin D deficiency, and disturbances in calcium-phosphorus metabolism. Changes in serum Ca and PHOS can also feedback-regulate the levels of FGF23, klotho, 1,25(OH)2D3, and iPTH (59). 1,25(OH)2D3, FGF23, and klotho may also interactively participate in the pathogenesis of DKD. Macrophage infiltration is a significant pathological feature of DKD and is closely related to the degree of kidney injury and interstitial fibrosis (60). FGF23 promotes the polarization of macrophages to the pro-inflammatory phenotype (M1), and klotho expression is significantly upregulated in M1 macrophages. In contrast, 1,25(OH)2D3 can stimulate M2 macrophages to increase Arg-1 expression, inhibiting FGF23-induced TNF-α upregulation, and participating in anti-inflammatory and anti-fibrotic responses. The balance of opposing effects between vitamin D and FGF23 may influence the trends of inflammation and fibrosis in DKD (61). Zou XR et al. discovered that Shenyuan granules can modulate vitamin D levels and improve kidney injury and calcium-phosphorus metabolism abnormalities in DKD mice by intervening in the synthesis of renal 24-hydroxylase and 1α-hydroxylase through the klotho/FGF23/Egr1 signaling pathway (62).

### 4.2 Clinical significance of bone metabolism markers

The results suggest that bone metabolism markers play a crucial role in DKD progression and may have significant clinical predictive value. ROC curve analysis shows that klotho, FGF23, 25(OH)D3, iPTH, Ca and PHOS each have predictive value for DKD occurrence, with potential applications in DKD diagnosis. The combined predictive model achieved an AUC of 0.846, highlighting the potential of these markers for improving prediction accuracy. Additionally, the AUC values for 25(OH)D3, iPTH, Ca and PHOS in predicting DKD severity were >0.85, showing high sensitivity and specificity. The combined model reached an AUC of 0.951, confirming its potential clinical utility. Previous studies have suggested that bone metabolism is closely related to women, but it is also a significant health issue for men (63). The gender differences in bone metabolism warrant further investigation.

### 4.3 Clinical applications and future research directions

These findings provide a valuable reference for managing bone metabolism in DKD patients. Monitoring klotho, FGF23, 25(OH)D3, iPTH, Ca and PHOS levels is crucial for early detection of DKD progression and assessing disease severity. The combined use of these markers could improve diagnostic accuracy and support personalized treatment. Future research should further explore the mechanisms of bone metabolism markers in DKD, especially their association with cardiovascular complications. Additionally, the impact of interventions targeting these markers on DKD progression needs further investigation. Such research could lead to new treatment strategies to improve DKD patient outcomes.

## 5 Limitations

This study has limitations including a small sample size, cross-sectional design restricting causal inference, insufficient consideration of all potential confounders, single-point measurement of bone metabolism markers, limited external validity, and lack of in-depth mechanistic studies. The study explored only certain bone metabolism indicators and did not include imaging assessments like bone density. Future research should expand the scope, validate results in larger and more diverse populations, and comprehensively evaluate the role of bone metabolism dysregulation in DKD progression.

## 6 Conclusion

The study indicates that changes in Klotho, FGF23, 25(OH)D3, iPTH, and Ca levels are closely related to DKD progression. These markers are significant for DKD diagnosis and prognosis and may serve as potential therapeutic targets. Further research should continue to explore the mechanisms and clinical applications of these markers to improve treatment outcomes and quality of life for DKD patients.

## Data Availability

The original contributions presented in the study are included in the article/supplementary material Further inquiries can be directed to the corresponding author.
